# Lipid accumulation product, poverty income ratio, and bone mineral density in U.S. adults: a mediation analysis based on NHANES (2009–2020)

**DOI:** 10.3389/fnut.2024.1466288

**Published:** 2024-10-03

**Authors:** Zihao Chen, Haobo Ye, Enli Li, Yuzhe Lin, Chen Jin, Lei Yang

**Affiliations:** Department of Orthopaedic Surgery, The Second Affiliated Hospital and Yuying Children Hospital of Wenzhou Medical University, Wenzhou, China

**Keywords:** lipid accumulation product, osteoporosis, bone mineral density, obesity, National Health and Nutrition Examination Survey

## Abstract

**Objective:**

This study aims to investigate the relationship between the lipid accumulation product (LAP) index and total femur bone mineral density (BMD), while also examining the mediating role of the poverty-income ratio (PIR).

**Methods:**

Using the most recent data from the National Health and Nutrition Examination Survey (NHANES) spanning 2009 to 2020, multivariate logistic regression models were employed in this study to investigate the relationship between the LAP index and total femur BMD. Saturation effects and potential non-linear associations were examined using a smooth curve-fitting approach to determine saturation levels. Interaction tests and subgroup analyses were also performed. Additionally, a mediation analysis was conducted to explore the mediating role of PIR.

**Results:**

Three thousand two hundred and twenty three participants aged 20 years or older were recruited for this study. Multivariate regression analysis demonstrated a greater total femur BMD in individuals with a high LAP index. Additionally, analysis of the saturation effect and smooth curve fitting identified a clear saturation effect between the LAP index and total femur BMD. A saturation value of 16.05 was determined when investigating the relationship between the LAP index and total femur BMD. Subgroup analysis revealed no significant interaction effects after adjusting for covariates. Moreover, mediation analysis indicated that the LAP index had a substantial direct effect on total femur BMD (*p* < 0.0001), with PIR partially mediating this relationship (1.115%, *p* = 0.0280).

**Conclusion:**

The results of this investigation demonstrated a saturation effect between the LAP index and total femur BMD, which may have been mediated by PIR.

## 1 Introduction

Osteoporosis, a systemic skeletal disorder that increases the risk of fractures, is characterized by a decrease in bone mass and the deterioration of bone tissue microstructure (1). The economy and public health are greatly impacted by osteoporosis as the global populace ages (2). This impact is largely driven by the increased healthcare costs associated with managing fractures, loss of mobility, and long-term care, which are common consequences of osteoporosis (3). Previous research has indicated an annual increase in the prevalence of osteoporosis among middle-aged and older adults (4, 5). This increase can be attributed to factors such as longer life expectancy, declining physical activity levels, and inadequate intake of calcium and vitamin D, which are critical for maintaining bone health (6). A decreased bone mineral density (BMD), a well-established marker of osteoporosis, is associated with an elevated risk of fractures (7). As a result, there is growing emphasis on identifying new risk factors for low bone mineral density in osteoporosis, which could lead to the development of new preventative strategies.

Obesity is defined as abnormal or excessive body fat and has a detrimental impact on health, being strongly associated with the onset of various chronic diseases (8). Obesity influences skeletal homeostasis in a complex manner, exerting both positive and negative effects on the development of osteoporosis. Although it was once widely accepted that obesity was positively associated with bone mass or density (9, 10), recent studies suggest a more nuanced relationship (11, 12). Body mass index (BMI) and waist circumference (WC) are currently the most widely used clinical markers for assessing obesity. Many experts consider BMI to be a relatively crude and controversial method for assessing the risk of various diseases and mortality, as it only reflects overall obesity and cannot account for other body components such as muscle mass, bone density, and fat distribution (13, 14). WC measures the extent of central or abdominal obesity, which contributes to metabolic abnormalities and increases the risk of diabetes, non-alcoholic fatty liver disease (NAFLD), and metabolic syndrome (MetS) (15). However, WC cannot differentiate between visceral adipose tissue (VAT) and subcutaneous adipose tissue (SAT); the former refers to ectopic fat accumulation, which leads to insulin resistance and organ dysfunction (16).

In the field of metabolic research, the lipid accumulation product (LAP) index has gained increasing attention. The LAP index, calculated by combining triglyceride (TG) levels and waist circumference (WC), is used to assess and reflect the extent of abdominal lipid accumulation (17). Compared to conventional lipid profiles, the LAP index demonstrates significant predictive power for chronic kidney disease, diabetes, and other related disorders (18–20). Compared to conventional measures such as waist-to-height ratio, BMI, and WC, the LAP index exhibits greater predictive efficiency (21, 22), and it has demonstrated strong predictive ability in in various clinical settings (23, 24). According to several studies, LAP may be a sign of NAFLD, heart disease, type 2 diabetes, and MetS (21, 25–27).

In addition, obesity (generally meaning high LAP index) is associated with higher incomes for people (28, 29), which may lead to a higher poverty-income ratio (PIR). On the other hand, Wang et al. employed multiple linear regression to examine the relationship between PIR and BMD in women, identifying a strong positive correlation (30). This association was further confirmed by a recent cross-sectional study conducted on adult men, which also highlighted the importance of socioeconomic status in managing osteoporosis (31). The relationship between socioeconomic status (SES) and health outcomes, including BMD and obesity, is well-documented (32). Low socioeconomic status (SES) is frequently associated with food insecurity, limited access to nutritious foods, and environments that discourage physical activity, all of which collectively contribute to obesity and poor bone health. Previous studies have highlighted that a lower poverty-income ratio (PIR) is associated with higher rates of obesity, due to increased exposure to food swamps—areas characterized by a high density of fast food outlets and limited access to fresh produce (33). Furthermore, poverty can limit opportunities for physical activity, a situation exacerbated by high crime rates and poorly designed built environments, both of which act as barriers to maintaining a healthy weight and preserving bone density (34). In light of these correlations, PIR is thought to mediate the link between BMD and the LAP index.

Currently, there is insufficient evidence to establish a connection between the LAP index and total femur BMD. The aim of our study was to address these knowledge gaps by investigating the association between the LAP index and femur BMD in adult U.S. participants, and by quantifying the role of PIR in mediating the relationship between the LAP index and total femur BMD.

## 2 Materials and methods

### 2.1 Study population

**Figure 1:**
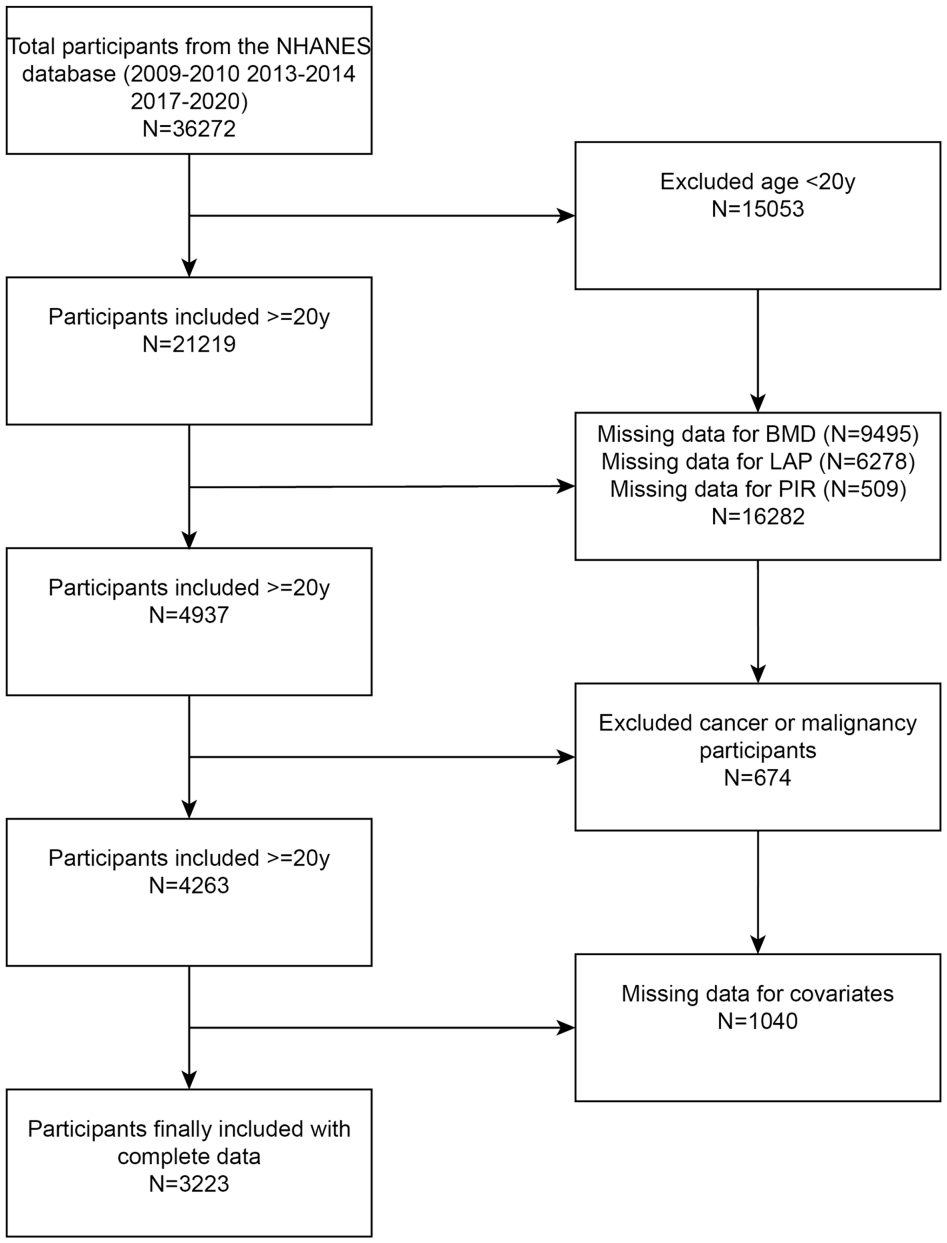
Flowchart illustrating participant selection. LAP, lipid accumulation product; BMD, bone mineral density; NHANES, National Health and Nutrition Examination Survey.

The National Health and Nutrition Examination Survey (NHANES) is an extensive, ongoing cross-sectional study conducted in the U.S., aimed at addressing emerging public health issues and collecting precise data on health-related topics. This study exclusively used data from NHANES, including laboratory components and interviews, to examine the relationship between diet and health in the U.S. The data for this investigation were drawn from NHANES 2009–2020; however, due to the unavailability of BMD data, NHANES 2011–2012 and NHANES 2015–2016 were excluded. According to the inclusion–exclusion criteria, 15,053 individuals under the age of 20 were excluded, 9,495 lacked BMD data, 6,278 lacked LAP data, 509 lacked PIR data, individuals with cancer or malignancies were excluded, and 1,040 lacked data on covariates. Ultimately, 3,223 participants were included in the study (Figure 1).

### 2.2 Exposure measurement and outcome ascertainment

The primary outcome of this investigation was the assessment of total femur BMD using dual-energy X-ray absorptiometry (DXA). The LAP score was calculated using the following formulas: [WC (cm) – 65] × [TG (mmol/L)] for males and [WC (cm) – 58] × [TG (mmol/L)] for females. TG and WC were measured in mmol/L and cm, respectively.

### 2.3 Covariates

To strengthen the correlation between the LAP index and total femur BMD, the following covariates were adjusted: race, gender, age, education level, smoking status, marital status, moderate physical activity, diagnosed diabetes, alcohol use, alkaline phosphatase (ALP), blood urea nitrogen (BUN), aspartate aminotransferase (AST), alanine aminotransferase (ALT), total calcium, creatinine, total bilirubin, phosphorus, total protein, high-density lipoprotein cholesterol (HDL), uric acid, and low-density lipoprotein cholesterol (LDL). Pre-specified effect modifiers were also applied to assess the impact of interaction, with stratification variables including gender (male/female), age (≤65/>65 years), education level, diabetes status (yes/no/borderline), smoking status (yes/no), and moderate physical activity (yes/no).

### 2.4 Statistical analysis

Sampling weights are often used in NHANES to take into account more complex study designs. Mean (standard deviation, [SD]) was used to report continuous data having a normal distribution. Frequencies (%) were used to display categorical variables. Applying the Pearson chi-square test for categorical data and the one-way ANOVA for continuous. We examined, both with and without adjusting for potential confounding variables, the relationship between the LAP index and total femur BMD using logistic regression models. The relationship between these two was investigated further using propensity score matching and several sensitivity analyses. The present study employed curve fitting techniques to investigate the potential association between the LAP index and the total femur BMD. The existence of a non-linear connection was demonstrated, and likelihood ratio tests were used to determine the values for inflection points. Subgroup analyses were then stratified using hierarchical logistic regression models according to factors such as gender, age, education level, diabetes, smoking status, and moderate activities. Parallel mediator analysis was used to determine the possible mediated influence of PIR on the relationship between the LAP index and total femur BMD. The impact of the LAP index on total femur BMD in the absence of mediators is known as the direct effect (DE). PIR’s indirect effects (IE), which are mediated by mediators, are the results of the total femur BMD. By dividing IE by TE (total effect), the proportion of mediators was calculated. Random forest (RF) analysis and Gradient Boosting Machine (GBM) model were used to analyze the importance of each feature affecting BMD. With Empower Stats and R, all statistical analyses were performed. A statistically significant two-tailed *p*-value was less than 0.05.

## 3 Results

### 3.1 Baseline features

A total of 3,223 participants qualified for inclusion in the study. Of these, 53.96% were men and 46.04% were women. The LAP index was treated both as a categorical variable (divided into tertiles) and as a continuous independent variable, with the lowest tertile serving as the reference group. Statistically significant differences were observed across the LAP index groups for race, gender, age, education level, smoking status, marital status, moderate physical activity, diagnosed diabetes, PIR, BUN, AST, ALP, ALT, creatinine, phosphorus, total bilirubin, uric acid, HDL-C, LDL-C, and total femur BMD (Table 1).

**Table 1:** Sample characteristics weighted for the research.

Lipid accumulation product	Quartile1 (<28.65)	Quartile2 (28.65–51.24)	Quartile3 (>51.24)	*p*-value
	*N* = 1,074	*N* = 1,074	*N* = 1,075	
**Age (year)**	49.2071 ± 15.5417	52.8810 ± 14.6358	54.5982 ± 14.1988	<0.00001
**Gender (%)**				0.000107
Male	46.5496	54.1247	54.8764	
Female	53.4504	45.8753	45.1236	
**Race (%)**				0.000027
Mexican American	5.4989	6.4520	10.4493	
Other Hispanic	5.4673	5.0194	5.6138	
Non-Hispanic White	69.1201	71.9058	70.5892	
Non-Hispanic Black	11.8511	10.5873	8.3251	
Other Race—Including Multi-Racial	8.0625	6.0355	5.0225	
**Education level (%)**				<0.00001
Less than 9th grade	3.5167	3.5161	6.1554	
9-11th grade	6.8089	11.0652	13.3152	
High school graduate	21.7774	22.2446	25.1986	
Some college or AA degree	29.3428	30.7266	31.6889	
College graduate or above	38.5543	32.4475	23.6419	
**Smoked at least 100 cigarettes (%)**				0.000005
Yes	41.9799	48.2988	52.6210	
No	58.0201	51.7012	47.3790	
**Marital status (%)**				0.001171
Married	58.0920	61.3720	59.9607	
Widowed	10.2813	9.6603	10.9673	
Divorced	8.9292	11.8869	12.1929	
Separated	1.6584	1.6766	1.7475	
Never married	14.5075	9.6879	9.7572	
Living with partner	6.5316	5.7163	5.3746	
**Moderate activities (%)**				<0.000001
Yes	54.6773	48.5876	40.8241	
No	45.3227	51.4124	59.1759	
**Diagnosed diabetes (%)**				<0.000001
Yes	4.7158	8.5050	14.4650	
No	93.7613	89.6025	81.3568	
Borderline	1.5229	1.8925	4.1783	
Waist circumstance (cm)	86.2251 ± 9.6434	99.0498 ± 10.5364	107.7061 ± 11.8730	<0.000001
Poverty income ratio	3.2406 ± 1.6520	3.1711 ± 1.6274	2.9750 ± 1.6394	0.000626
ALT (U/L)	23.1565 ± 20.5628	24.0101 ± 12.6123	27.7943 ± 18.5392	<0.000001
ALP (U/L)	62.7386 ± 21.9448	68.6091 ± 21.7309	71.5357 ± 23.2008	<0.000001
AST (U/L)	26.4441 ± 25.7685	23.9077 ± 10.9429	26.1755 ± 19.3150	0.003986
Blood urea nitrogen (mmol/L)	4.8332 ± 1.7122	4.9071 ± 1.6786	5.0741 ± 1.9803	0.006952
Creatinine (μmol/L)	76.7053 ± 24.0306	78.8579 ± 21.7308	79.9936 ± 35.4379	0.020079
Phosphorus (mmol/L)	1.1972 ± 0.1722	1.1601 ± 0.1699	1.1550 ± 0.1771	<0.000001
Total bilirubin (μmol/L)	12.7845 ± 5.5178	12.1097 ± 5.0105	11.7371 ± 4.9141	0.000014
Total calcium (mmol/L)	2.3422 ± 0.0821	2.3440 ± 0.0859	2.3411 ± 0.0914	0.745916
Total protein (g/L)	70.4962 ± 4.6029	70.6573 ± 4.3504	70.6294 ± 4.5430	0.671579
Uric acid (μmol/L)	290.0038 ± 73.0938	328.2033 ± 75.4063	352.2107 ± 81.1241	<0.000001
Triglyceride (mmol/L)	0.7469 ± 0.2637	1.1186 ± 0.3279	1.7128 ± 0.5366	<0.000001
HDL-C (mmol/L)	1.6984 ± 0.4723	1.4504 ± 0.3562	1.2180 ± 0.3025	<0.000001
LDL-C (mmol/L)	2.7283 ± 0.8044	3.0717 ± 0.9003	3.1294 ± 0.9458	<0.000001
Past 12 months how often drink alcoholic beverage	5.9428 ± 27.6333	4.3060 ± 15.8671	6.6375 ± 51.6131	0.273775
Total femur bone mineral density (g/cm^2^)	0.9203 ± 0.1534	0.9610 ± 0.1605	0.9929 ± 0.1501	<0.000001

For continuously varying variables, mean ± SD: By using a weighted linear regression model, the *p*-value was computed.

% is a category variable: Using the weighted chi-square test, the *p*-value was determined.

### 3.2 Association between lipid accumulation product index and total femur BMD

**Figure 2:**
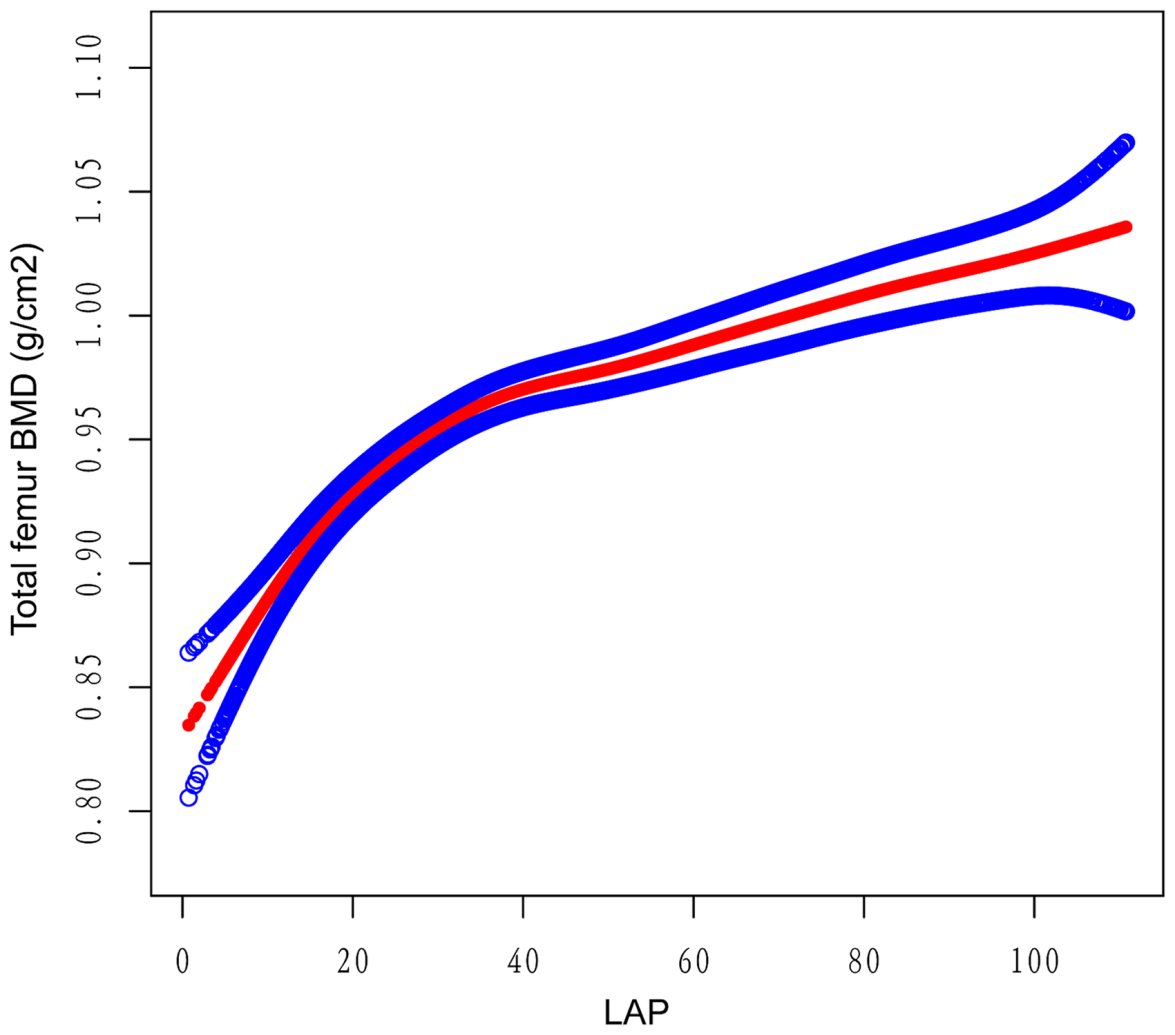
The relationship between the lipid accumulation product index and the total bone mineral density of the femur. The smooth red line indicates the best possible fit of the curve between the variables. The blue shading indicates the 95% CI for the fit. All potential confounds were eliminated.

Multivariate regression analysis revealed a positive association between the LAP index and total femur BMD, as shown in Models 1 [*β* (95% CI) = 0.0012 (0.0010, 0.0014)], 2 [β (95% CI) = 0.0014 (0.0012, 0.0016)], and 3 [β (95% CI) = 0.0013 (0.0010, 0.0015)]. When the LAP index was categorized into tertiles, individuals in the highest tertile had significantly greater total femur BMD compared to those in the lowest tertile. This was demonstrated by Models 1 [0.0727 (0.0594, 0.0859)], 2 [0.0817 (0.0702, 0.0932)], and 3 [0.0706 (0.0569, 0.0844)]. Based on Table 2, none of the three models demonstrated any significant trends (*p* > 0.05). After adjusting for all covariates, a non-linear association between the LAP index and total femur BMD was assessed using piecewise linear regression and smooth curve fitting. The total femur BMD exhibited a curvilinear increase in relation to the LAP index, eventually reaching a plateau at a specific threshold (Figure 2). To determine this LAP index turning point value, piecewise linear regression was used. For LAP index values below 16.05, each unit increase was associated with a 0.0092 g/cm^2^ increase in total femur BMD. However, the upward trend in total femur BMD significantly slowed when the LAP index exceeded 16.05. A log-likelihood ratio test was performed, yielding a significance level of <0.001, confirming that the saturation point was 16.05 (Table 3).

**Table 2:** Association of LAP with total femur BMD.

	Crude model (Model 1)	Minimally adjusted model (Model 2)	Fully adjusted model (Model 3)
	β (95%CI) *p* value	β (95%CI) *p* value	β (95%CI) *p* value
LAP	0.0012 (0.0010, 0.0014) <0.000001	0.0014 (0.0012, 0.0016) <0.000001	0.0013 (0.0010, 0.0015) <0.000001
**LAP (tertile)**			
Q1	Reference	Reference	Reference
Q2	0.0407 (0.0278, 0.0537) <0.000001	0.0440 (0.0329, 0.0552) <0.000001	0.0396 (0.0278, 0.0513) <0.000001
Q3	0.0727 (0.0594, 0.0859) <0.000001	0.0817 (0.0702, 0.0932) <0.000001	0.0706 (0.0569, 0.0844) <0.000001
*P* for trend	<0.000001	<0.000001	<0.000001

Note: Model 1: No adjustment for covariates.

Model 2: Race, gender, and age were adjusted.

Model 3: Race, gender, age, education level, smoking status, marital status, moderate activities, diagnosed diabetes, alcoholic use, blood urea nitrogen, Aspartate aminotransferase, Alkaline phosphatase, Alanine aminotransferase, creatinine, phosphorus, total bilirubin, total calcium, total protein, uric acid, high-Density Lipoprotein Cholesterol, low-Density Lipoprotein Cholesterol were adjusted.

**Table 3:** Analysis of the LAP saturation effect and the total BMD of the femur (g/cm^2^).

Model	Total femur BMD
	Adjustedβ (95%CI) *p* value
**Model 1**	
The standard linear mode	0.0013 (0.0010, 0.0015) <0.0001
**Model 2**	
Turning point (K)	16.05
LAP < 16.05	0.0092 (0.0069, 0.0115) <0.0001
LAP > 16.05	0.0011 (0.0008, 0.0013) <0.0001
Log likelihood ratio test	<0.001

All models were adjusted for race, gender, age, education level, smoking status, marital status, moderate activities, diagnosed diabetes, alcoholic use, blood urea nitrogen, Aspartate aminotransferase, Alkaline phosphatase, Alanine aminotransferase, creatinine, phosphorus, total bilirubin, total calcium, total protein, uric acid, HDL-C, LDL-C were adjusted.

### 3.3 Subgroup analysis

**Figure 3:**
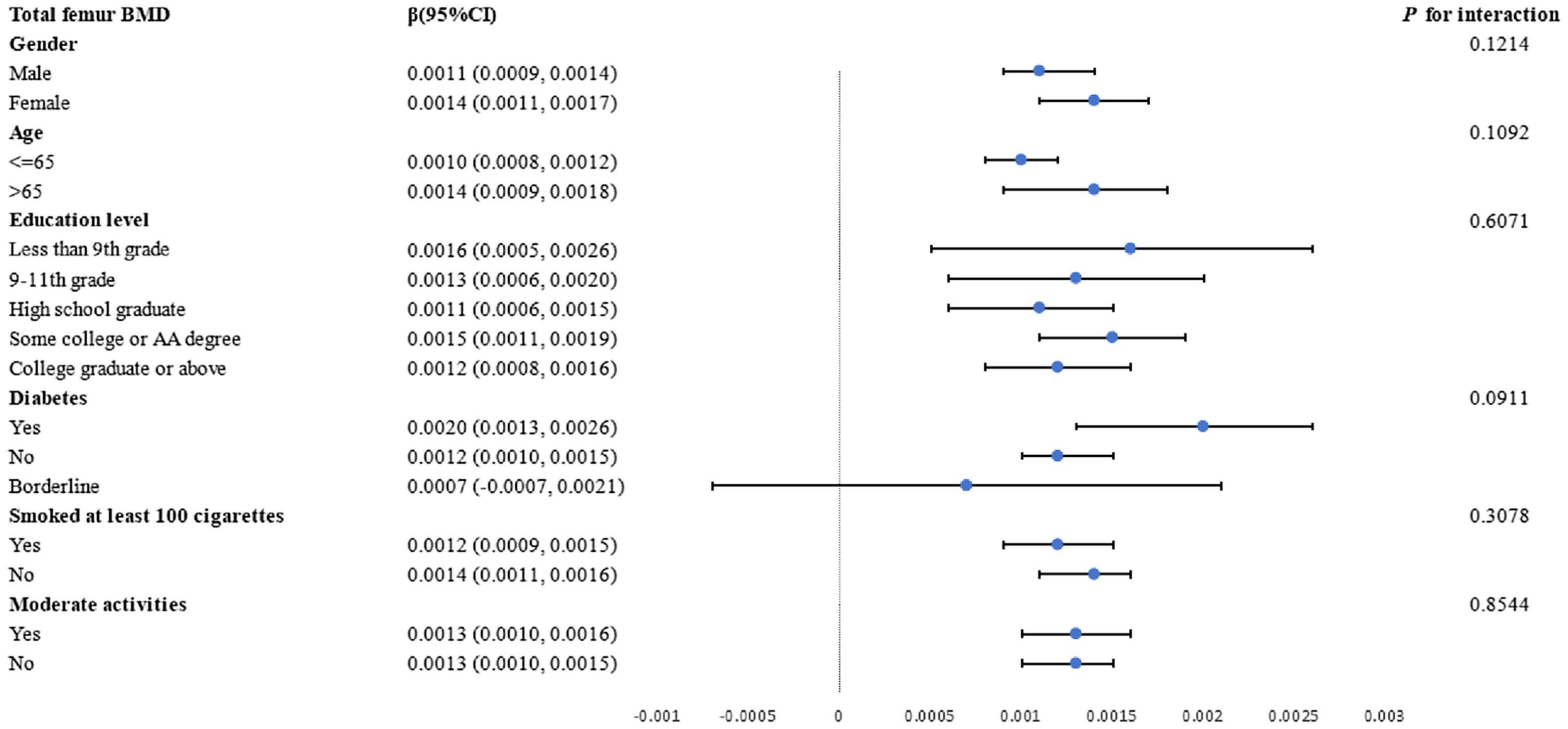
Analysis of LAP index subgroups in relation to total femur BMD.

Subgroup analyses by gender, age, education level, diabetes status, and smoking status were conducted to further assess the strength of the association between the LAP index and femur BMD. As shown in Figure 3, a positive correlation between total femur BMD and the LAP index was observed. The stratifications mentioned above did not affect this relationship, and all interactions with *p* values less than 0.05 were statistically significant.

### 3.4 Mediation analysis

**Figure 4:**
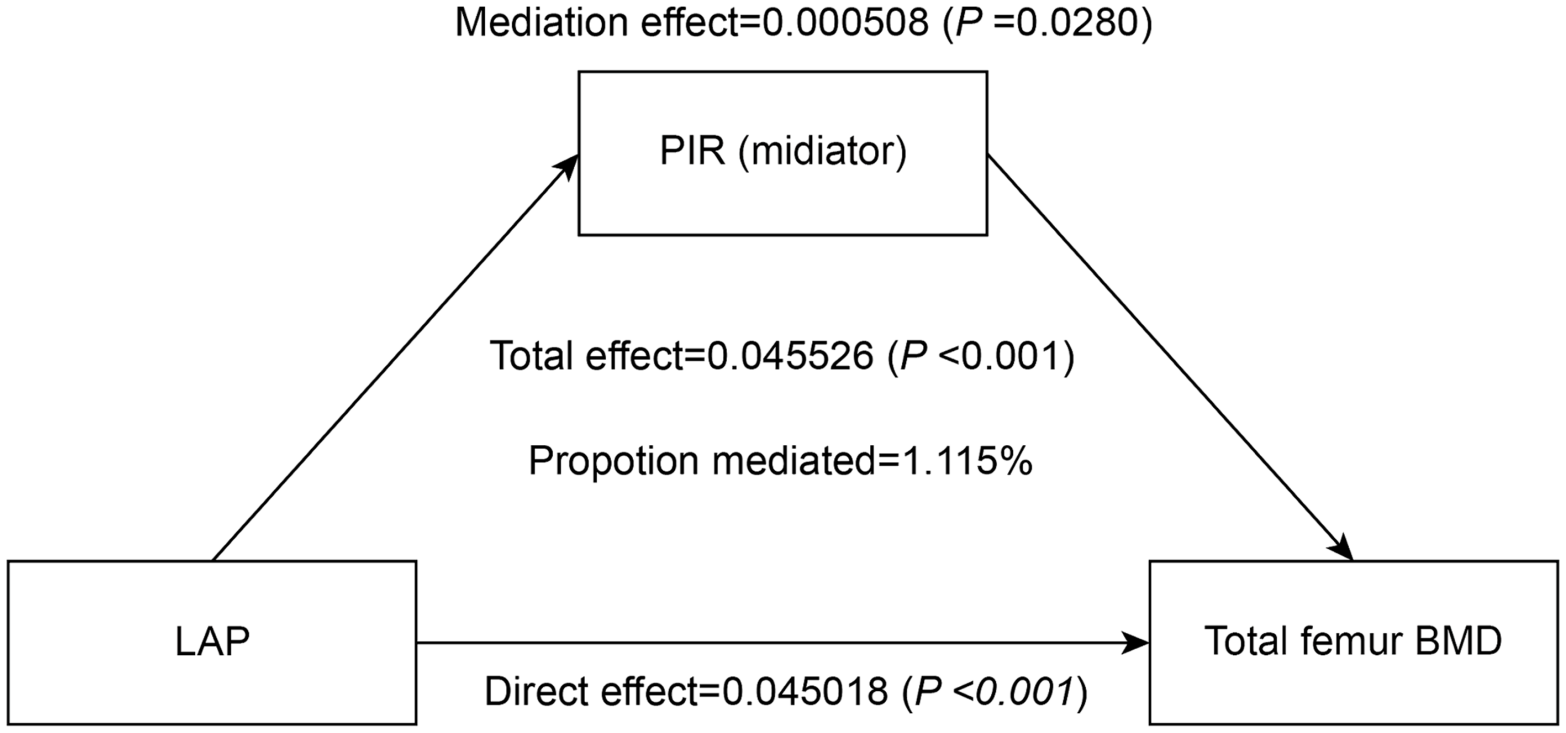
Effect of the PIR (mediators) on the relationship between the LAP index (exposure) and total femur BMD (outcome).

To assess the extent to which PIR mediated the association between the LAP index and total femur BMD, mediation analyses were conducted. After adjustments made in Model III, our study revealed an intriguing finding: the LAP index had a substantial direct effect on total femur BMD (*p* < 0.0001), while PIR exhibited a partial mediation effect in this relationship (*p* = 0.0280). Notably, our calculations indicated that PIR mediated 1.115% of the complex association between the LAP index and total femur BMD (Table 4; Figure 4).

**Table 4:** Ratio of family income to poverty as a mediator in the associations of LAP with total femur BMD (g/cm^2^).

Mediation effect (LAP-PIR-total femur BMD)	Estimate	95% CI lower	95% CI upper	*p*-value
Total effect	0.045526	0.031963	0.048655	<0.0001
Mediation effect	0.000508	0.000072	0.001431	0.0280
Direct effect	0.045018	0.031110	0.047901	<0.0001
Proportion mediated	0.011150	0.001746	0.036441	0.0280

All models were adjusted for race, gender, age, education level, smoking status, marital status, moderate activities, diagnosed diabetes, alcoholic use, blood urea nitrogen, Aspartate aminotransferase, Alkaline phosphatase, Alanine aminotransferase, creatinine, phosphorus, total bilirubin, total calcium, total protein, uric acid, HDL-C, LDL-C were adjusted.

### 3.5 Machine learning

**Figure 5:**
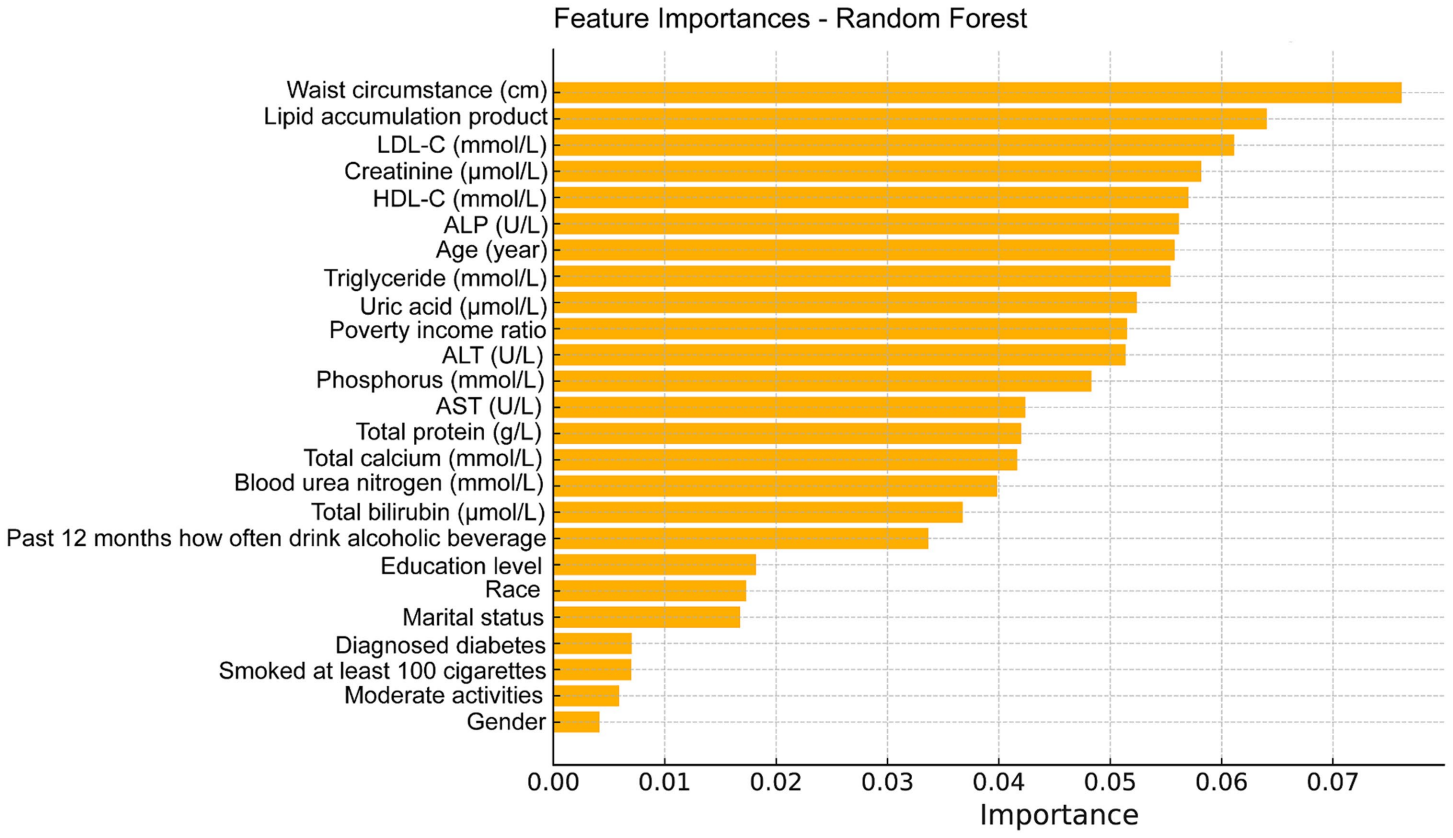
Feature importance maps of relative contributions to predicting total femur BMD were generated by random forest regression models.

**Figure 6:**
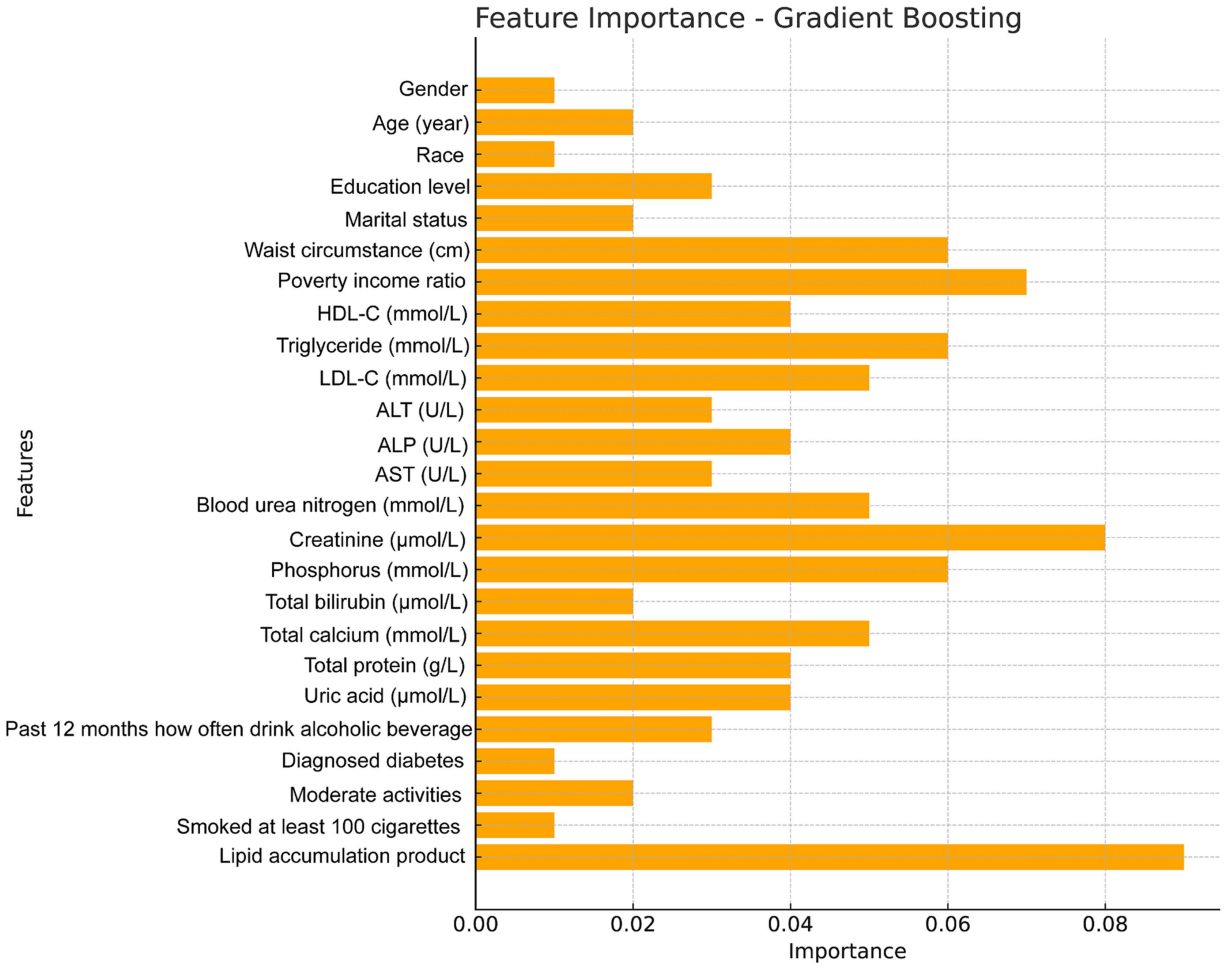
Feature importance maps of relative contributions to predicting total femur BMD were generated by gradient boosting models.

The feature importance plot generated from the RF regression model highlights the relative contribution of various clinical and demographic factors in predicting total femur BMD. This plot clearly illustrates that clinical measures related to fat distribution and lipid metabolism are among the most critical predictors of bone density, with the LAP index being a significant factor in the model and a key indicator in this study (Figure 5). Additionally, we utilized the GBM model to analyze these factors and obtained results similar to those of the random forest analysis, further confirming that the LAP index is the most important factor in the model (Figure 6).

## 4 Discussion

This investigation of 3,223 participants demonstrated a positive, nonlinear correlation between the LAP index and total femur BMD. Notably, the total femur BMD for all participants revealed a LAP index saturation point of 16.05. Beyond this threshold, the rate of increase in total femur BMD naturally declined, which is crucial for maintaining BMD at an optimal level. Consequently, the current study identifies the LAP index as an appropriate metric for clinically assessing total femur BMD. To our knowledge, this is the first study to demonstrate a saturation effect between baseline LAP index and total femur BMD levels.

Osteoporosis and obesity have become a global epidemic, but the link between the two is unclear. Osteoporosis is a biological disorder that weakens and increases the brittleness of bones over time, increasing the chance that they may fracture (35). One essential marker for osteoporosis diagnosis is a low BMD. Epidemiological research indicates that as the global population ages, lifestyle changes, economic growth, osteoporosis, and inadequate bone density are becoming increasingly common. According to reports, osteoporosis affects more than one-third of older adults in the United States, and its prevalence is steadily increasing (36). With regard to the skeletal system as a whole, a study has shown that body fat is one of the most significant predictors of BMD (31). Several metrics are used to assess obesity, particularly given the known harmful effects of intra-abdominal fat. BMI and WC are globally accepted standards for defining obesity (37–40). We found that BMI was positively associated with BMD in numerous studies (41, 42). That being said, some studies indicate that WC, a metric used to assess abdominal obesity, is negatively correlated with BMD (43), backed up by relevant research (44–46). Furthermore, several studies have shown that VAT is negatively correlated with BMD (47–50). Additionally, Kim et al. found that triglycerides were negatively correlated with BMD in both men and women (51). Despite mounting evidence from several epidemiological studies showing that traditional anthropometric parameters are related to BMD, the obesity conundrum remains unresolved. Therefore, identifying an obesity indicator that addresses the obesity paradox is crucial. Furthermore, although WC and BMI are commonly used as standard measures to describe obesity, they cannot differentiate between lean and fat tissue. Because of doubts about the reliability of these traditional indicators, researchers have recently developed new adiposity indices (52, 53). The LAP index was developed to provide a comprehensive measure that accounts for anatomical and physiological changes associated with visceral fat accumulation (25, 26, 54). Kahn is credited with developing the LAP index, a marker for assessing excessive lipid accumulation (21). In a cross-sectional study, Taverna et al. demonstrated that the LAP index showed a high level of accuracy in diagnosing MetS (55). The findings of Shi et al.’s study supported the notion that arterial stiffness is positively correlated with the LAP index (56). The study by Kim et al. revealed a strong correlation between the LAP index and both hyperuricemia and cardiovascular risk, compared to other adiposity measures such as the visceral adiposity index and body roundness index (57). Additionally, methods such as computed tomography and magnetic resonance imaging are frequently used for the sensitive detection of visceral fat. However, these techniques have several drawbacks, including high costs, lengthy procedures, and potential radiation risks. These methods are therefore impractical for extensive population screens. In contrast to conventional lipid profiles, LAP may evaluate a wide range of metabolic disease syndromes and offer a thorough evaluation of an individual’s metabolic health (58–60). Our investigation identified a saturation association between the LAP index and total femur BMD, the breakpoint at 16.05 indicates a substantial threshold influence.

The mechanisms underlying the observed positive correlation between BMD and the LAP index are not yet fully understood. However, this relationship may be explained by several hypothesized mechanisms. Firstly, obese or overweight individuals may produce higher levels of insulin, estrogen, and other endocrine hormones, which inhibit bone remodeling and resorption, thereby helping to maintain bone mass (61–64). Secondly, the accumulation of excess fat can alter the mechanical properties of the skeleton, leading to changes in bone tissue (65, 66). Thirdly, activation of the Wnt/*β*-catenin signaling pathway has been shown to effectively inhibit adipocyte proliferation and differentiation, leading to reduced visceral fat deposition (67). The enhancement of osteoblast function depends on the successful initiation and activation of the Wnt/*β*-catenin signaling pathway, with mutations in the LRP5 gene within this pathway recognized as a significant contributor to the development of osteoporosis (68). Thus, it is reasonable to suggest that a high LAP index or severe obesity may influence the development of osteoporosis by interfering with the Wnt/β-catenin signaling pathway. Lastly, obesity may promote the differentiation of bone mesenchymal stem cells into adipocytes, increasing the number of adipocytes and reducing the number of osteoblasts (69).

The results of this investigation demonstrated a non-linear relationship between the LAP index and total femur BMD, both before and after applying a generalized additive model to account for confounding factors. This study is notable for being the first to identify threshold and saturation effects, as well as a non-linear relationship between the LAP index and total femur BMD. These findings provide medical professionals with additional tools to help obese patients maintain a healthy LAP index (around 16.05), support optimal bone mineral density, and reduce the risk of obesity-related illnesses and complications. However, further research is needed to better understand the mechanisms behind the LAP index’s saturation effects on BMD. VAT plays a crucial role in the relationship between the LAP index and BMD (70–72). VAT is metabolically active and has been associated with increased inflammation, which can negatively affect bone health and lead to lower BMD (73). As the LAP index is an indicator of VAT, it is reasonable to hypothesize that individuals with a LAP > 16 have higher VAT levels, and consequently, a higher risk of reduced BMD. However, it is also noteworthy that obesity, characterized by a higher LAP index, has paradoxically been associated with increased BMD. This suggests a complex interplay in which the protective effects of mechanical loading from increased body weight may be offset by the detrimental effects of VAT-induced inflammation. In addition, the differential impact of VAT and SAT on BMD provides insight into the results observed at the LAP index turning point (74). As the LAP index increases, indicating higher VAT, one might expect a corresponding decrease in BMD due to the inflammatory and metabolic effects of VAT. However, not all obese individuals have high levels of VAT; some may predominantly have SAT, which could explain the observed variation in BMD at similar LAP levels (75). This complexity suggests that the LAP index is an imperfect proxy for VAT and that the relationship between adiposity, fat distribution, and bone health is multifaceted. The results indicating a turning point in the relationship between the LAP index and BMD could be partially explained by the differential distribution of fat tissue in obese individuals. While a higher LAP index generally reflects higher VAT, which is associated with lower BMD, the presence of significant SAT may mitigate this effect. This variability in fat distribution among individuals with similar LAP values could account for the observed inconsistencies in BMD outcomes (76). Therefore, further research is necessary to explore the nuances of fat distribution and its specific impact on bone health. Another factor contributing to the LAP index saturation effects is the presence of a unique bone-fat axis that exists between bone and adipose tissue *in vivo*, along with various bioactive compounds that help maintain bone homeostasis (77). In addition, we conducted a mediation analysis that found PIR partially mediated the relationship between a high LAP index and increased bone mineral density. In recent years, researchers have conducted several studies on the relationship between PIR and BMD. Data from the Louisiana Osteoporosis Study suggest that SES and BMD are positively correlated in the general population (78). Additionally, Xiao et al. demonstrated that PIR is positively associated with total spine BMD (31). A meta-analysis of eight epidemiological studies supports the hypothesis that individuals with higher income levels are more likely to have greater BMD, as evidenced by the majority of population-based research (79). This cross-sectional study, which included 3,223 representative U.S. subjects, similarly corroborated this conclusion. Given this research, it is believed that PIR may have partially mediated the positive correlation between the LAP index and total femur BMD, and the findings of this study’s mediation analysis lend support to this notion. This mediation can be explained through several social mechanisms. For example, individuals with lower PIR are more likely to live in food swamps, which increases the risk of obesity due to the high availability of energy-dense, nutrient-poor foods. Additionally, low PIR is associated with higher crime rates, which can limit outdoor physical activity and further contribute to obesity and poor bone health. These factors highlight the complex interplay between socioeconomic status (SES), environmental factors, and health outcomes (32–34).

The sample size is large and representative, which is one of the key strengths of this research. The study also carefully adjusted for several confounding variables, ensuring that the results are reliable and applicable to a broad population. Additionally, by employing the LAP index, this study effectively explores the relationship between the LAP index and clinical outcomes. However, it is important to acknowledge certain limitations of the present research. The primary issue is that it remains uncertain whether the observed decrease in femur bone mineral density was directly caused by the LAP index. Furthermore, this study cannot fully rule out residual confounding from unknown factors, even after adjusting for several likely confounders. Moreover, we acknowledge that PIR, as a standalone socioeconomic indicator, has limitations in accurately reflecting an individual’s specific nutritional status. More comprehensive indicators are needed to better assess the nutritional status of individuals. Lastly, while a broad range of study populations was included in this research, the findings may not be applicable to certain populations—such as cancer patients—since they were not part of the current study.

## 5 Conclusion

We identified a curvilinear relationship, more specifically a saturation effect, between the LAP index and total femur BMD in U.S. adults. According to the present study, individuals over the age of 20 may be able to achieve an optimal LAP/BMD balance by maintaining a modest LAP index (around 16.05), which would promote healthy bone formation. Moreover, the positive correlation between the LAP index and total femur BMD is partially mediated by PIR. Looking ahead, the LAP index offers a simple and affordable tool to help obese individuals maintain optimal BMD and reduce their risk of obesity-related illnesses.

## Data Availability

Publicly available datasets were analyzed in this study. This data can be found here: https://www.cdc.gov/nchs/nhanes/index.htm.
